# Serological and Molecular Surveillance for SARS-CoV-2 Infection in Captive Tigers (*Panthera tigris*), Thailand

**DOI:** 10.3390/ani12233350

**Published:** 2022-11-29

**Authors:** Nareerat Sangkachai, Somjit Chaiwattanarungruengpaisan, Metawee Thongdee, Parut Suksai, Siriporn Tangsudjai, Peerawat Wongluechai, Sarin Suwanpakdee, Witthawat Wiriyarat, Ruangrat Buddhirongawatr, Luxsana Prasittichai, Anurux Skulpong, Pilailuk Akkapaiboon Okada, Pilaipan Puthavathana, Weena Paungpin

**Affiliations:** 1 The Monitoring and Surveillance Center for Zoonotic Diseases in Wildlife and Exotic Animals, Faculty of Veterinary Science, Mahidol University, Nakhon Pathom 73170, Thailand; 2 Department of Clinical Sciences and Public Health, Faculty of Veterinary Science, Mahidol University, Nakhon Pathom 73170, Thailand; 3 Protected Area Regional Office 3 (Ban Pong), Ratchaburi 70110, Thailand; 4 Wildlife Rescue Center III (Khao Prathap Chang), Ratchaburi 70110, Thailand; 5 National Institute of Health, Department of Medical Sciences, Nonthaburi 11000, Thailand; 6 Center for Research and Innovation, Faculty of Medical Technology, Mahidol University, Nakhon Pathom 73170, Thailand

**Keywords:** captive tigers, SARS-CoV-2, plaque reduction neutralization test, surveillance, Thailand

## Abstract

**Simple Summary:**

SARS-CoV-2 can infect a variety of domestic and wild animals worldwide. Therefore, tigers are a susceptible species and potential viral reservoirs. It is important to investigate COVID-19 disease exposure in these endangered animals. This study tested for SARS-CoV-2 neutralizing antibodies in 62 serum samples from captive tigers in Ratchaburi province, western Thailand during 2020–2021. In addition, nine swab samples were also collected from tigers with a history of exposure to COVID-19 patients; however, SARS-CoV-2 RNA was not detected in any of the swab samples. SARS-CoV-2 neutralizing antibodies against the Delta variant were found in samples collected from four zoo tigers in November 2021 when that variant was circulating widely in Thailand. In addition, cross-neutralization against Wuhan Hu-1 could be observed in all of the seropositive tiger serum samples while a low-level tier of neutralizing antibodies against the Omicron BA.2 subvariant could be found in only one seropositive tiger. The source of SARS-CoV-2 infection in these tigers most likely came from close contact with the infected animal caretakers who engaged in activities such as tiger petting and feeding. To the authors’ knowledge, this is the first case of natural SARS-CoV-2 infection in captive tigers during the COVID-19 outbreak in Thailand. We suggest that continuous surveillance of SARS-CoV-2 infection in captive tigers should be performed to prevent and control outbreaks of COVID-19 zoonotic disease.

**Abstract:**

Coronavirus disease (COVID-19) is an emerging infectious disease caused by SARS-CoV-2. Given the emergence of SARS-CoV-2 variants, continuous surveillance of SARS-CoV-2 in animals is important. To monitor SARS-CoV-2 infection in wildlife in Thailand, we collected 62 blood samples and nine nasal- and rectal-swab samples from captive tigers (*Panthera tigris*) in Ratchaburi province in Thailand during 2020–2021. A plaque reduction neutralization test (PRNT) was employed to detect SARS-CoV-2 neutralizing antibodies. A real-time RT-PCR assay was performed to detect SARS-CoV-2 RNA. Our findings demonstrated that four captive tigers (6.5%, 4/62) had SARS-CoV-2 neutralizing antibodies against Wuhan Hu-1 and the Delta variant, while no SARS-CoV-2 RNA genome could be detected in all swab samples. Moreover, a low-level titer of neutralizing antibodies against the Omicron BA.2 subvariant could be found in only one seropositive tiger. The source of SARS-CoV-2 infection in these tigers most likely came from close contact with the infected animals’ caretakers who engaged in activities such as tiger petting and feeding. In summary, we described the first case of natural SARS-CoV-2 infection in captive tigers during the COVID-19 outbreak in Thailand and provided seroepidemiological-based evidence of human-to-animal transmission. Our findings highlight the need for continuous surveillance of COVID-19 among the captive tiger population and emphasize the need to adopt a One Health approach for preventing and controlling outbreaks of COVID-19 zoonotic disease.

## 1. Introduction

The 2019 coronavirus (COVID-19) pandemic, caused by the severe acute respiratory syndrome coronavirus 2 (SARS-CoV-2), originated in the Wuhan province of China in late 2019. The COVID-19 pandemic has caused significant global impacts resulting in more than six hundred million confirmed cases and six and a half million deaths as of 1 October 2022 [1]. SARS-CoV-2 is shown to be associated with ancestor viruses from wildlife, in particular horseshoe bats (*Rhinolophus affinis*) or Malayan pangolins (*Manis javanica*) [2,3]. SARS-CoV-2 mainly circulates in the human population and is being sustained through human-to-human transmission. Moreover, multiple animals have been reported to be infected with SARS-CoV-2 [4]. The emergence of SARS-CoV-2 variants still raises concerns about zoonotic (animal-to-human) and reverse-zoonotic (human-to-animal) infection of SARS-CoV-2 between humans and animals. It is important to understand SARS-CoV-2 epidemiology to prevent the risk of infection/re-infection of humans and animals and requires a robust One Health-based investigation.

Humans are the major sources of SARS-CoV-2 infection in domestic and captive animals. Transmission of SARS-CoV-2 from humans to animals had been reported in a cat, dog, pet ferret, mink, lion, tiger, puma and white-tailed deer [5,6,7,8,9,10,11]. Information on SARS-CoV-2 infection in domestic and zoo animals that live closely with humans is still growing. Infection in various host species could result in genetic adaptation of the SARS-CoV-2 genome and increase the risks of the SARS-CoV-2 pandemic.

Captive feline species in zoos such as tigers and lions are susceptible to SARS-CoV-2 infection [12]. Surveillance studies in different countries have documented cases of natural SARS-CoV-2 infection through contact with people infected with SARS-CoV-2. The first confirmed cases have been reported in Malayan tigers (*Panthera tigres jacksoni*), Amur tigers (*P. tigres altaica*) and African lions (*P. leo krugeri*) at Bronx Zoo, New York, USA [13]. Tigers and lions were infected with different clades of SARS-CoV-2. A neutralizing antibody titer of 1:64 was found in one tiger [13]. The persistence of viral RNA fecal shedding in these animals was observed for up to 35 days after the cessation of respiratory signs [14]. In India, several Asiatic lions (*P. leo persica*) at different zoological parks were infected with the Delta variant (B.1.617.2) [15,16], and SARS-CoV-2 neutralizing antibody titers of 1:64–1:256 were observed in these lions [15]. In Spain, four lions (*P. leo*) were infected with the SARS-CoV-2 variant (B.1.177) at Barcelona Zoo. SARS-CoV-2 neutralizing antibody responses lasted at least four months [17]. In South Africa, three African lions were infected with the Delta variant at a private zoo in Johannesburg. One lion developed pneumonia while the other cases had mild infection. Those animals remained positive for SARS-CoV-2 RNA for up to 7 weeks [18]. The source of infection in most of these feline cases was presumably acquired by asymptomatically infected zookeepers who tested positive for SARS-CoV-2. Therefore, the transmission of the virus from infected humans to these animals through close contact has been presumed.

As of 14 September 2022, Thailand had approximately 4.6 million confirmed COVID-19 cases with 32,564 deaths [19]. To date, cats and dogs have been infected during the five epidemic waves by different SARS-CoV-2 variants, suggesting human to animal transmission in COVID-19-positive households [20,21,22,23]. Contrary to companion animals, captive wild animals are still under-explored for SARS-CoV-2 detection. Thus, the current investigation aimed to provide epidemiological data on surveillance of SARS-CoV-2 infection in captive tigers in Thailand.

## 2. Materials and Methods

### 2.1. Ethical Approval

All analysis of animals and viruses were conducted under the approval of the Institute for Animal Care and Use Committee, Faculty of Veterinary Science, Mahidol University (MUVS-2020-08-33 and MUVS-2022-01-01) and the approval of the Institutional Biosafety Committee of Mahidol University (IBC#2022-048). Written informed consent was obtained from animal owners and animal keepers to collect animal behavior data as well as the relevant data involving epidemiological risk factors. Data collection through interviewing was reviewed and approved by the Institutional Review Board of Mahidol University (MU-CIRB 2022/035.0804).

### 2.2. Study Population and Sample Collection

A total of 62 tiger (*P. tigris*) sera used in this study were collected during the years 2020–2021 by veterinarians under cooperation between the Department of National Parks, Wildlife and Plant Conservation and the Monitoring and Surveillance Center for Zoonotic Diseases in Wildlife and Exotic Animals, Faculty of Veterinary Science, Mahidol University. All adult tigers were living in Ratchaburi province, western Thailand. Demographic data related to the captive tigers, including habitat locations and sex of the animals, are shown in Table 1. During the time of sample collection, the animals did not exhibit clinical symptoms associated with respiratory disease. A physical examination was performed on each anesthetized animal in order to assess their overall health status. Blood collection from the saphenous vein of the individual animals was carried out for laboratory examinations. Whole blood was allowed to clot prior to 4 °C storage. Serum separation from the clotted blood was conducted on the same day, and the serum samples were stored at −20 °C until testing. Moreover, nasal and rectal swab samples were collected from nine tigers with a history of exposure to a SARS-CoV-2 infected person. Each individual swab was placed into a tube containing 1 mL of viral transport medium (VTM). All swab samples were maintained at 4 °C during transportation to the laboratory.

### 2.3. Virus and Cell Culture Work

African green monkey kidney epithelial (Vero: ATCC, CCL-81) cells were cultured in Eagle’s minimum essential medium (EMEM, Gibco, Grand Island, NY, USA) containing 200 IU/mL of penicillin, 200 µg/mL of streptomycin, 75 µg/mL of gentamicin sulfate and 6 µg/mL of amphotericin B supplemented with 10% fetal bovine serum (FBS) at 37 °C with 5% CO_2_.

The SARS-CoV-2 viruses used in the plaque reduction neutralization test (PRNT) for neutralizing antibodies detection comprised SARS-CoV-2/human/THA/MUMT-4/2020 (GenBank accession number MT781414: Wuhan Hu-1), hCoV-19/Thailand/Nan_SEQ7413/2021 (GISAID accession number EPI_ISL_3797061: Delta variant) and hCoV-19/Thailand/NIC_BKK_SEQ4804/2022 (GISAID accession number EPI_ISL_9611330: Omicron BA.2 subvariant). These viruses were primarily isolated from COVID-19 patients and propagated in Vero cells. Viral stocks were prepared from the fourth–fifth passage in Vero cells in which the viral titers were determined by a plaque assay. All the infection experiments were performed in a biosafety level-3 (BLS-3) laboratory.

### 2.4. Plaque Reduction Neutralization Test (PRNT) for SARS-CoV-2 Neutralizing Antibody Detection

Briefly, an equal volume (130 µL) of serial two-fold dilutions of heat-inactivated serum (56 °C, 30 min) and SARS-CoV-2 at 800 plaque-forming units (PFU)/mL were mixed and then incubated at 37 °C for 1 h with 5% CO_2_. The serum–virus mixture (250 µL) was transferred into wells of pre-formed Vero cell monolayers (3.5 × 10^5^ cells/well) and incubated at 37 °C with 5% CO_2_ for 1 h. The cell monolayer was then overlaid with 3% carboxymethylcellulose (CMC) in cell culture medium and incubated for 3 days, at which time the plates were fixed and stained. A neutralizing antibody titer was defined as the reciprocal of the highest serum dilution resulting in plaque reduction of at least 80% (PRNT80) [6,24,25]. Positive and negative serum control were obtained from a COVID-19 vaccinated individual and a non-COVID-19 vaccinated individual, respectively. Geometric mean titer (GMT) of the virus strain was calculated. A neutralizing antibody titer < 10 was assigned as 5, while a neutralizing antibody titer ≥ 320 was assigned as 320. Neutralizing antibody titers ≥ 20 were considered as seropositive and indicated previous infection. Archived tiger serum samples (n = 20) from the pre-COVID-19 cohort in 2019 were included for testing in the PRNT. The PRNT procedure was performed in the BSL-3 facility.

### 2.5. RNA Isolation and SARS-CoV-2 Genome Detection

Total RNA was extracted from each nasal and rectal swab using the total RNA mini kit (Geneaid Biotech Ltd., New Taipei City, Taiwan) according to the manufacturer’s procedure. Viral RNA was detected by the real-time RT-PCR format using 16220F (5′ CGCATACAGTCTTRCAGGCT 3′), 16353R (5′ GTGTGATGTTGAWATGACATGGTC 3′) and 16276P (5′ FAM-TTAAGATGTGGTGCTTGCATACGTAGAC-lABkFQ 3′) and targeting the RNA-dependent RNA polymerase/helicase (*RdRp/Hel*) gene [26]. The real-time RT-PCR mixture contained 5 μL of template RNA, 10 µL of 2× reaction mix, 0.2 µM of each primer, 0.1 µM of probe, 0.4 μL of SuperScript™ III RT/Platinum™ *Taq* mix (Invitrogen, Waltham, MA, USA) and 0.4 μL of ROX™ reference dye. Nuclease-free water was added up to 20 µL. Positive control (RNA transcripts derived from recombinant plasmids containing a partial *RdRp/Hel* gene insert of the Delta variant isolate) and negative control (nuclease-free water) were included in each run. PCR reaction was incubated at 50 °C for 15 min and 95 °C for 3 min, followed by 45 cycles of denaturation at 95 °C for 15 sec and annealing/extension at 55 °C for 30 s. A cycle threshold (Ct) of <38 was considered positive.

### 2.6. Statistical Analysis

Data management of the investigated tigers was performed using Microsoft Office Excel 2019. The GMT (95% CI) was statistically analyzed using GraphPad Prism version 8.0 (Graphpad Software Inc., San Diego, CA, USA). The prevalence of SARS-CoV-2 infection in captive tigers was demonstrated as a percentage by dividing the number of positive results by the total number of tested.

## 3. Results

**Figure 1:**
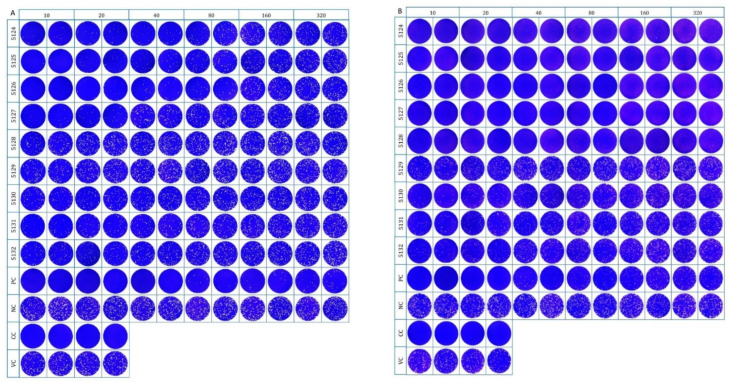
The plaque reduction neutralization test of nine tiger serum samples (ID 5124–5132) for SARS-CoV-2 neutralizing antibodies against (**A**) Wuhan Hu-1 and (**B**) the Delta variant. All samples were two-fold serially diluted and were tested in duplicate. PC: vaccinated human serum; NC: non-vaccinated human serum; CC: Vero cell control; VC: virus control.

SARS-CoV-2 neutralizing antibodies were found in samples collected from four captive tigers living in a private zoo in November 2021. The PRNT result demonstrated that 6.5% (4/62) of the tiger serum samples had neutralizing antibodies against both Wuhan Hu-1 and the Delta variant. Neutralizing antibody titers against Wuhan Hu-1 and the Delta variant ranged from 1:20 to 1:40 and from 1:80 to 1:160, respectively (Table 2, Figure 1). Among the positive serum samples, the GMTs (95% CI) of the neutralizing antibody titers against Wuhan Hu-1 and the Delta variant were 28.28 (11.63–48.37) and 69.64 (19.27–176.70), respectively. The PRNT results demonstrated the cross-neutralizing antibodies of the tiger serum samples against SARS-CoV-2 Wuhan Hu-1 and the Delta variant. In addition, archived tiger serum samples (n = 20) from the pre-COVID-19 cohort in 2019 were also included in the PRNT. None of these serum samples tested positive for SARS-CoV-2-neutralizing antibodies against both Wuhan Hu-1 and the Delta variant.

**Figure 2:**
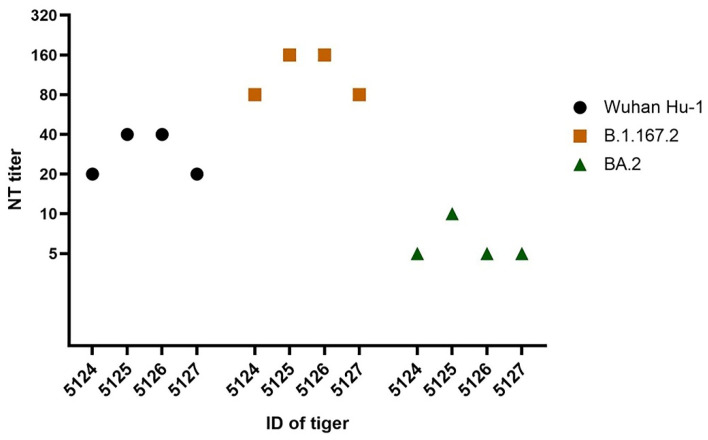
Neutralizing antibody titers against strains of SARS-CoV-2 in four individual seropositive tigers. NT: titer-neutralizing antibody titer; ID: identity number.

To determine whether the cross-neutralizing antibodies in seropositive tigers provide a protective immune response against new SARS-CoV-2 variants, we selected the Omicron BA.2 subvariant as the test antigen used in the PRNT. Our result demonstrated that only one tiger, with the identity number (ID) 5126, had neutralizing antibody titers of 1:10 while no neutralizing antibody titers were found in the other three tigers (Table 2, Figure 2).

SARS-CoV-2 seropositive samples were only found in female tigers which were uniformly five years old. None of the four female and five male tigers at the zoo showed respiratory signs at the time of physical health checks. In addition, all of the zoo tigers were collected for swab samples to detect SARS-CoV-2 RNA. Both nasal and rectal swabs were negative for SARS-CoV-2 virus genome detection, suggesting the absence of active SARS-CoV-2 infection in the tested animals at the time of sampling.

All four female tigers were housed in the same area and separated from the other five male tigers. Aggressive behavior was obviously observed in the male group rather than the female group. Prior to our visit, the zoo was temporarily closed in response to the COVID-19 situation. However, the zoo workers continued the essential activities to ensure the well-being of the animals. Animal keepers undertook daily tasks, including food preparation and feeding of the tigers. No zoo workers used any personal protective equipment and only washed their hands before and after work. Close contact between workers and female tigers included feeding, petting, cuddling and enrichment activities. However, there was less contact between the workers and the male tigers due to their aggressive behavior. Approximately two months before our visit, three animal keepers who took care of the tigers were found to be positive for SARS-CoV-2 with mild clinical symptoms. Therefore, the possible exposure of SARS-CoV-2 to the female tigers was potentially considered through close contact with the animal keepers who had a recent history of SARS-CoV-2 infection.

## 4. Discussion

The natural infection of SARS-CoV-2 has been reported in 26 animal species worldwide, including domestic and wild animals [27]. The majority of cases of natural infection of SARS-CoV-2 in animals have been linked to an infected human [27]. The spillover of virus from infected humans to animals could result in the genetic adaptation of the SARS-CoV-2 genome leading to potential emergence of novel SARS-CoV-2 variants. There is evidence showing that some of the new SARS-CoV-2 variants could potentially escape the immune system and may affect the efficacy of the current vaccines [28,29,30,31,32,33]. Evolutionary changes in viruses occur after cross-species transmission and could possibly alter the pathogenicity and transmissibility of a virus in new host species [2,10,34]. Thus, it is important to investigate SARS-CoV-2 exposure among animal species in order to identify potential animal hosts and their roles in the maintenance of the virus. In the present study, we aimed to serosurvey captive tigers in Thailand for neutralizing antibodies to SARS-CoV-2. The data from our seroepidemiological study add to the growing body of information on SARS-CoV-2 infection in wildlife.

Based on epidemiological and molecular studies, Thailand was first attacked by SARS-CoV-2 (Wuhan Hu-1) in March 2020, and until now, we have experienced five waves of the different SARS-CoV-2 variants. At the time we found seropositive captive tigers in November 2021, we faced the fourth wave of the SARS-CoV-2 outbreak, primarily driven by the Delta variant [35]. It was reported that the Delta variant is approximately twice as transmissible as the original Wuhan strain from China [36]. There were nearly two million confirmed COVID-19 human cases and twenty thousand deaths by 11 November 2021 [37]. In the present study, neutralizing antibodies with high titers (range 1:80–1:160) against the Delta variant detected among the captive tigers were consistent with the circulating SARS-CoV-2 variant in the human population at the same period of time, although the presence of an active infection in those tigers was undetected. Data collection through interviewing animal owners and animal keepers provided information on animal behavior as well as the relevant data involving epidemiological risk factors which could help us to identify the possible route of virus transmission. The source of the Delta variant infection in the tigers most likely came from close contact with the infected caretakers who engaged in activities such as animal feeding, petting, cuddling and animal enrichment. Nevertheless, it could not be excluded that other routes of transmission may be possible. All four female seropositive tigers were housed in the same area and separated from the other five male seronegative tigers. It could not be excluded that tiger-to-tiger transmission did occur. Tiger-to-tiger transmission was demonstrated by Grome et al. [38].

The PRNT is the gold standard when determining the level of neutralizing antibody titers which influences the protective immune response. Seropositivity was defined based on the PRNT80 titer values which represent the reciprocal of the highest serum dilution at which plaque numbers are reduced by at least 80% relative to virus control. The PRNT cut-off value for a positive result was ≥1:20. This assay cut-off value has also been utilized by other studies [6,24,25]. Our serological investigation using the GMT (95% CI) showed that the four positive tigers (6.5%, 4/62) had neutralizing antibodies against Wuhan Hu-1 and the Delta variant of 28.28 (11.63–48.37) and 69.64 (19.27–176.70), respectively. Although at lower titers, the detectable neutralizing antibodies against Wuhan Hu-1 in the four positive tigers implied the cross-neutralizing antibody response of the natural SARS-CoV-2 infection in the seropositive tigers. Given the emergence of new SARS-CoV-2 variants, the presence of the protective antibody found in SARS-CoV-2 virus-infected animals needs to be addressed. Our study demonstrated that one out of four seropositive tigers had a detectable neutralizing antibody titer of 1:10 to the Omicron BA.2 subvariant. The results implied that immunological protection from a previous Delta variant infection in tigers may not protect against a new Omicron BA.2 subvariant infection. Several studies have reported the lower capacity to neutralize the Omicron subvariants by human sera from vaccine recipients induced by the current generation COVID-19 vaccines [28,29,30,31,32,33].

The role of tigers in SARS-CoV-2 maintenance and transmission within species or across species remains unclear. However, the introduction of SARS-CoV-2 to wildlife could result in the formation of animal reservoirs. The spread of SARS-CoV-2 in animal populations not only affects the health of the infected populations but also probably facilitates the emergence of new virus variants [10,39]. Epidemiologic investigation and surveillance monitoring for SARS-CoV-2 infection in animals are important and require One Health sectors to support the activities, especially when the spread of the virus is not totally under control. Adopting a One Health approach is critical to prevent outbreaks of zoonotic diseases as well as facilitate an understanding of ecological and epidemiological dynamics of the diseases.

## 5. Conclusions

Our results demonstrated the detection of neutralizing antibodies against the SARS-CoV-2 Delta variant in captive tigers in a zoo during the fourth wave of the COVID-19 outbreak in Thailand. Cross-neutralization against Wuhan Hu-1 could be observed in the seropositive tiger serum samples. The history of close contact with animal keepers who had tested positive for SARS-CoV-2 suggested the possible route of transmission. Thus, public awareness of SARS-CoV-2 infection in wild animals, especially in close contact with COVID-19 cases, should be raised to reduce the potential risks at the human–wild animal interface. Moreover, continuous monitoring and surveillance systems for SARS-CoV-2 in wild animals are needed to identify potential animal hosts and their roles in the maintenance of the virus.

## Figures and Tables

**Table 1:** Demographic characteristics of samples derived from captive adult tigers during 2020–2021, Ratchaburi province, Thailand.

Sample Collection		Place	No. of Tigers		No. of Samples	
Year	Date		Female	Male	Serum	Swab
2020	29 May	WBC No.1	25	10	35	NA
	21–22 October	WBC No.2	8	1	9	NA
2021	26 April	WBC No.2	8	-	8	NA
	19 August	WBC No.1	-	1	1	NA
	9 November	Zoo	4	5	9	9 N, 9 R
Total			45	17	62	18

Abbreviations: wildlife breeding center: WBC; nasal swab: N; rectal swab: R; not available: NA.

**Table 2:** Neutralizing antibody titers against strains of SARS-CoV-2 in seropositive tigers.

Seropositive Tigers		Neutralizing Antibodies Titer ^a^		
ID	Sex	Wuhan Hu-1	Delta (B.1.617.2)	Omicron (BA.2)
5124	female	20	80	<10
5125	female	40	160	<10
5126	female	40	160	10
5127	female	20	80	<10
NC	female	<10	<10	<10
PC	female	160	20	20

Abbreviations: identity number: ID; negative serum control: NC; positive serum control: PC. ^a^: A neutralizing antibody titer was defined as the reciprocal of the highest serum dilution resulting in plaque reduction of at least 80% (PRNT80) compared to the virus control.

## Data Availability

The data presented in the study are available in the manuscript.

## References

[1] WHO Coronavirus (COVID-19) Dashboard. https://covid19.who.int/.

[2] Andersen K.G., Rambaut A., Lipkin W.I., Holmes E.C., Garry R.F. (2020). The proximal origin of SARS-CoV-2. Nat. Med..

[3] Zhang T., Wu Q., Zhang Z. (2020). Probable Pangolin Origin of SARS-CoV-2 Associated with the COVID-19 Outbreak. Curr. Biol..

[4] SARS-CoV-2 in animals situation report. https://www.woah.org/app/uploads/2022/07/sars-cov-2-situation-report-14.pdf.

[5] Calvet G.A., Pereira S.A., Ogrzewalska M., Pauvolid-Correa A., Resende P.C., Tassinari W.S., Costa A.P., Keidel L.O., da Rocha A.S.B., da Silva M.F.B. (2021). Investigation of SARS-CoV-2 infection in dogs and cats of humans diagnosed with COVID-19 in Rio de Janeiro, Brazil. PLoS ONE.

[6] Patterson E.I., Elia G., Grassi A., Giordano A., Desario C., Medardo M., Smith S.L., Anderson E.R., Prince T., Patterson G.T. (2020). Evidence of exposure to SARS-CoV-2 in cats and dogs from households in Italy. Nat. Commun..

[7] Račnik J., Kočevar A., Slavec B., Korva M., Rus K.R., Zakotnik S., Zorec T.M., Poljak M., Matko M., Rojs O.Z. (2021). Transmission of SARS-CoV-2 from Human to Domestic Ferret. Emerg. Infect. Dis..

[8] Fenollar F., Mediannikov O., Maurin M., Devaux C., Colson P., Levasseur A., Fournier P.E., Raoult D. (2021). Mink, SARS-CoV-2, and the Human-Animal Interface. Front. Microbiol..

[9] Hale V.L., Dennis P.M., McBride D.S., Nolting J.M., Madden C., Huey D., Ehrlich M., Grieser J., Winston J., Lombardi D. (2022). SARS-CoV-2 infection in free-ranging white-tailed deer. Nature.

[10] Tan C.C.S., Lam S.D., Richard D., Owen C.J., Berchtold D., Orengo C., Nair M.S., Kuchipudi S.V., Kapur V., van Dorp L. (2022). Transmission of SARS-CoV-2 from humans to animals and potential host adaptation. Nat. Commun..

[11] Farag E.A., Islam M.M., Enan K., El-Hussein A.M., Bansal D., Haroun M. (2021). SARS-CoV-2 at the human-animal interface: A review. Heliyon.

[12] Mathavarajah S., Dellaire G. (2020). Lions, tigers and kittens too: ACE2 and susceptibility to COVID-19. Evol. Med. Public Health.

[13] McAloose D., Laverack M., Wang L., Killian M.L., Caserta L.C., Yuan F., Mitchell P.K., Queen K., Mauldin M.R., Cronk B.D. (2020). From people to Panthera: Natural SARS-CoV-2 infection in tigers and lions at the Bronx Zoo. mBio.

[14] Bartlett S.L., Diel D., Wang L., Zec S., Laverack M., Martins M., Caserta L.C., Killian M.L., Terio K., Olmstead C. (2021). SARS-CoV-2 infection and longitudinal fecal screening in Malayan tigers (*Panthera Tigris Jacksoni*), Amur tigers (*Panthera Tigris Altaica*), and African lions (*Panthera Leo Krugeri*) at the Bronx Zoo, New York, USA. J. Zoo Wildl. Med..

[15] Karikalan M., Chander V., Mahajan S., Deol P., Agrawal R.-K., Nandi S., Rai S.-K., Mathur A., Pawde A., Singh K.-P. (2021). Natural infection of Delta mutant of SARS-CoV-2 in Asiatic lions of India. Transbound. Emerg. Dis..

[16] Mishra A., Kumar N., Bhatia S., Aasdev A., Kanniappan S., Sekhar A.T., Gopinadhan A., Silambarasan R., Sreekumar C., Dubey C.K. (2021). SARS-CoV-2 Delta variant among Asiatic lions, India. Emerg. Infect. Dis..

[17] Fernandez-Bellon H., Rodon J., Fernandez-Bastit L., Almagro V., Padilla-Sole P., Lorca-Oro C., Valle R., Roca N., Grazioli S., Trogu T. (2021). Monitoring Natural SARS-CoV-2 Infection in Lions (*Panthera leo*) at the Barcelona Zoo: Viral Dynamics and Host Responses. Viruses.

[18] Koeppel K.N., Mendes A., Strydom A., Rotherham L., Mulumba M., Venter M. (2022). SARS-CoV-2 Reverse zoonoses to pumas and lions, South Africa. Viruses.

[19] WHO Thailand Weekly Situation Update No. 247. https://cdn.who.int/media/docs/default-source/searo/thailand/2022_09_14_tha-sitrep-247-covid-19.pdf?sfvrsn=d1b0bfca_1.

[20] Jairak W., Charoenkul K., Chamsai E., Udom K., Chaiyawong S., Bunpapong N., Boonyapisitsopa S., Tantilertcharoen R., Techakriengkrai N., Surachetpong S. (2021). First cases of SARS-CoV-2 infection in dogs and cats in Thailand. Transbound. Emerg. Dis..

[21] Udom K., Jairak W., Chamsai E., Charoenkul K., Boonyapisitsopa S., Bunpapong N., Techakriengkrai N., Amonsin A. (2021). Serological survey of antibodies against SARS-CoV-2 in dogs and cats, Thailand. Transbound. Emerg. Dis..

[22] Jairak W., Charoenkul K., Chamsai E., Udom K., Chaiyawong S., Hangsawek A., Waenkaew S., Mungaomklang A., Tangwangvivat R., Amonsin A. (2022). Survey of SARS-CoV-2 in dogs and cats in high-risk areas during the second wave of COVID-19 outbreak, Thailand. Zoonoses Public Health.

[23] Jairak W., Chamsai E., Udom K., Charoenkul K., Chaiyawong S., Techakriengkrai N., Tangwangvivat R., Suwannakarn K., Amonsin A. (2022). SARS-CoV-2 delta variant infection in domestic dogs and cats, Thailand. Sci. Rep..

[24] Schulz C., Martina B., Mirolo M., Müller E., Klein R., Volk H., Egberink H., Gonzalez-Hernandez M., Kaiser F., von Köckritz-Blickwede M. (2021). SARS-CoV-2-specific antibodies in domestic cats during first COVID-19 wave, Europe. Emerg. Infect. Dis..

[25] Smith S.L., Anderson E.R., Cansado-Utrilla C., Prince T., Farrell S., Brant B., Smyth S., Noble P.M., Pinchbeck G.L., Marshall N. (2021). SARS-CoV-2 neutralising antibodies in dogs and cats in the United Kingdom. CRVIRO.

[26] Chan J.F., Yip C.C., To K.K., Tang T.H., Wong S.C., Leung K.H., Fung A.Y., Ng A.C., Zou Z., Tsoi H.W. (2020). Improved molecular diagnosis of COVID-19 by the novel, highly sensitive and specific COVID-19-RdRp/Hel real-time reverse transcription-polymerase chain reaction assay validated in vitro and with clinical specimens. J. Clin. Microbiol..

[27] Zhai S.L., Li C.L., Sun M.F., Zhang J.F., Zheng C., Liao M. (2022). Natural infections of SARS-CoV-2 increased in animals: How should humans interact with animals?. J. Med. Virol..

[28] Wibmer C.K., Ayres F., Hermanus T., Madzivhandila M., Kgagudi P., Oosthuysen B., Lambson B.E., de Oliveira T., Vermeulen M., van der Berg K. (2021). SARS-CoV-2 501Y.V2 escapes neutralization by South African COVID-19 donor plasma. Nat. Med..

[29] Tada T., Zhou H., Dcosta B.M., Samanovic M.I., Chivukula V., Herati R.S., Hubbard S.R., Mulligan M.J., Landau N.R. (2022). Increased resistance of SARS-CoV-2 Omicron variant to neutralization by vaccine-elicited and therapeutic antibodies. EBioMedicine.

[30] Hachmann N.P., Miller J., Collier A.-R.Y., Ventura J.D., Yu J., Rowe M., Bondzie E.A., Powers O., Surve N., Hall K. (2022). Neutralization Escape by SARS-CoV-2 Omicron Subvariants BA.2.12.1, BA.4, and BA.5. N. Engl. J. Med..

[31] Evans J.P., Zeng C., Qu P., Faraone J., Zheng Y.M., Carlin C., Bednash J.S., Zhou T., Lozanski G., Mallampalli R. (2022). Neutralization of SARS-CoV-2 Omicron sub-lineages BA.1, BA.1.1, and BA.2. Cell Host Microbe.

[32] Yu J., Collier A.Y., Rowe M., Mardas F., Ventura J.D., Wan H., Miller J., Powers O., Chung B., Siamatu M. (2022). Neutralization of the SARS-CoV-2 Omicron BA.1 and BA.2 Variants. N. Engl. J. Med..

[33] Lu L., Mok B.W.Y., Chen L.L., Chan J.M.C., Tsang O.T.Y., Lam B.H.S., Chuang V.W.M., Chu A.W.H., Chan W.M., Ip J.D. (2022). Neutralization of Severe Acute Respiratory Syndrome Coronavirus 2 Omicron Variant by Sera From BNT162b2 or CoronaVac Vaccine Recipients. Clin. Infect. Dis..

[34] Bashor L., Gagne R.B., Bosco-Lauth A.M., Bowen R.A., Stenglein M., VandeWoude S. (2021). SARS-CoV-2 evolution in animals suggests mechanisms for rapid variant selection. Proc. Natl. Acad. Sci. USA.

[35] Delta Variant Responsible for over 98 Percent of New Infections. https://www.nationthailand.com/in-focus/40008034.

[36] How Dangerous Is the Delta Variant (B.1.617.2)?. https://asm.org/Articles/2021/July/How-Dangerous-is-the-Delta-Variant-B-1-617-2.

[37] WHO Thailand Weekly Situation Update No. 209. https://cdn.who.int/media/docs/default-source/searo/thailand/2021_11_11_eng-sitrep-209-covid-19.pdf?sfvrsn=92f99090_3.

[38] Grome H.N., Meyer B., Read E., Buchanan M., Cushing A., Sawatzki K., Levinson K.J., Thomas L.S., Perry Z., Uehara A. (2022). SARS-CoV-2 Outbreak among Malayan Tigers and Humans, Tennessee, USA, 2020. Emerg. Infect. Dis..

[39] Meekins D.A., Gaudreault N.N., Richt J.A. (2021). Natural and Experimental SARS-CoV-2 Infection in Domestic and Wild Animals. Viruses.

